# Comparing Measures Of Functional Difficulty With Self-Identified Disability: Implications For Health Policy

**DOI:** 10.1377/hlthaff.2022.00395

**Published:** 2022-10

**Authors:** Jean P. Hall, Noelle K. Kurth, Catherine Ipsen, Andrew Myers, Kelsey Goddard

**Affiliations:** University of Kansas, Lawrence, Kansas.; University of Kansas.; University of Montana, Missoula, Montana.; University of Montana.; University of Kansas.

## Abstract

The Affordable Care Act mandated data collection standards to identify people with disabilities in federal surveys to better understand and address health disparities within this population. Most federal surveys use six questions from the American Community Survey (ACS-6) to identify people with disabilities, whereas many international surveys use the six-item Washington Group Short Set (WG-SS). The National Survey on Health and Disability (NSHD), which focuses on working-age adults ages 18–64, uses both question sets and contains other disability questions. We compared ACS-6 and WG-SS responses with self-reported disability types. The ACS-6 and WG-SS failed to identify 20 percent and 43 percent, respectively, of respondents who reported disabilities in response to other NSHD questions (a broader WG-SS version missed 4.4 percent of respondents). The ACS-6 and the WG-SS performed especially poorly in capturing respondents with psychiatric disabilities or chronic health conditions. Researchers and policy makers must augment or strengthen federal disability questions to improve the accuracy of disability prevalence counts, understanding of health disparities, and planning of appropriate services for a diverse and growing population.

Documenting the number of Americans with disabilities is essential for allocating sufficient resources and developing and maintaining programs to meet their needs.^1,2^ Equally important is a clear understanding of the types of disabilities experienced so that resources and programs can be tailored appropriately. Disability, however, is difficult to measure because it is not necessarily a static individual trait that can be uniformly identified with a single definition or survey question. According to the World Health Organization’s International Classification of Functioning, Disability, and Health, disability is a complex interaction of multiple factors including health condition, body function, environmental factors, and personal factors, which shape opportunity for activities and participation.^3^ Despite the importance of tracking disability, it is difficult to do so accurately because of variations across these factors and limits on the number of questions included in population-based surveys.^4^ Overall, the population of people with and causes of disability are diverse and complex, which makes population measurement challenging.

Despite these challenges, the Affordable Care Act (ACA) mandated that all federally funded national population health surveys include a uniform set of questions to identify respondents with disabilities to increase understanding of health disparities in the population.^5,6^ Before the ACA mandate, federal surveys used varied and inconsistent disability questions that captured different groups, making comparisons across surveys difficult.^7^ The uniform disability questions selected for use by most federal surveys are the six-item set from the Census Bureau’s American Community Survey (ACS-6), which ask respondents about difficulties hearing; seeing; concentrating, remembering, and making decisions; walking; dressing and bathing; and doing errands. Another question set, the Washington Group Short Set on Functioning (WG-SS), is considered an international standard and is used in the National Health Interview Survey, the National Health and Nutrition Examination Survey, and others to facilitate international comparisons.^8^

## Overview Of Disability Measures

### AMERICAN COMMUNITY SURVEY SIX-ITEM SET

The ACS-6 contains six yes-or-no questions about difficulty seeing; hearing; remembering, concentrating, and making decisions; walking; self-care; and doing errands. It is currently used in more than a dozen national surveys administered through various US federal departments and agencies, including the Census Bureau, and the Departments of Education, Labor, Housing and Urban Development, and Justice.^5^

The first iteration of ACS-6 questions was developed and tested in the 1990s and focused on the presence of disabling conditions. These questions were later criticized because they did not align with the social model of disability, which frames disability not only as an individual attribute but also as a result of societal barriers, such as inaccessible physical environments and negative attitudes.^3^ Within the social model, disability is manifested in terms of functional difficulties, and the current ACS-6 question set, revised in 2008, reflects this orientation.

In 2011 a report commissioned by the Department of Health and Human Services (HHS)^6^ recommended further analyses of the ACS-6 to better understand its limitations. Research since that time has shown that the ACS-6 has several drawbacks to measuring disability reliably. Specifically, it incorrectly counts people with temporary difficulties and systematically misses or undercounts certain subgroups of people with enduring disabilities.^9–12^ For instance, Bryce Ward and colleagues^12^ explored the consistency of responses to the ACS-6 with data from the Census Bureau’s Current Population Survey and found that more than half of respondents who responded “yes” to one or more ACS-6 questions did so inconsistently over time. They further found that changes in health status were associated with changes in disability reporting, highlighting that some people reported transitory, rather than enduring, difficulties.^11^ Similarly, Catherine Ipsen and colleagues^10^ found that among a group of more than 2,000 high school–age youth with disabilities qualifying for federal disability assistance, more than one-quarter with psychiatric disabilities and 20 percent with developmental disabilities did not respond “yes” to any of the ACS-6 questions, indicating that a large proportion of people with disabling conditions might not be counted by the ACS-6.

Data from federal surveys using the ACS-6 inform a wide range of state and federal programs and funding decisions, including assessment of health care access and health disparities, housing needs and housing fund allocations, planning for disaster response, and documenting discrimination in education and employment.^2^ Thus, if certain disability groups are consistently underrepresented using the ACS-6, their experiences will likewise be misdocumented, and they may receive insufficient attention by both federal and state programs.

### WASHINGTON GROUP SHORT SET ON FUNCTIONING

Similar to the ACS-6, the WG-SS includes six questions to capture functioning in the domains of seeing, hearing, walking, remembering and concentrating, self-care, and communication. The WG-SS was conceptualized during the 2001 International Seminar on the Measurement of Disability with the goal of developing a common set of questions that could be used in national surveys and compared internationally.^13^ The WG-SS has also been found to undercount many people with disabilities.^14^ Eric Lauer and colleagues^15^ reported significant differences in the prevalence of functional disability rates between the ACS-6 and WG-SS and called for additional research to compare these measures. Specifically, they found that various classifications using the WG-SS response set resulted in prevalence rates that were either significantly higher or lower than in the ACS-6.

### FILLING IN THE GAPS

To more fully understand and document the nature of disabilities captured and missed by the ACS-6 and WG-SS, we compared responses to these measures with several alternative questions on disability, using the National Survey on Health and Disability (NSHD; administered by several of the authors).^16^ The NSHD, which is focused on working-age adults with disabilities, includes the ACS-6 and WG-SS question sets. In addition, it asks respondents to both describe their disability with an open-ended question and select one of seven categories to characterize their primary condition. Thus, the NSHD provides a unique opportunity to compare how the same people responded to both function-based and condition-based questions. Findings from these analyses inform our recommendations for measuring disability in future national surveys. This research is particularly timely, given the need for accurate measurement of an emerging group of people with disabilities who experience physical and mental impairments attributable to long COVID.^17,18^

## Study Data And Methods

We used data from the second wave of the NSHD (*N* = 2,175), a national, internet-based survey of adults ages 18–64 with self-reported disabilities fielded from October 2019 to January 2020. Participants were recruited through more than seventy disability organizations, conferences, and meetings and through Amazon’s Mechanical Turk, an online crowdsourcing tool that can be used to screen and recruit survey takers.^16,19^ The NSHD was designed to collect information about respondents’ health, health insurance status, and access to health care services.^20,21^ The online survey platform was fully accessible to screen readers, and respondents had the option to complete the survey by telephone or proxy. The University of Kansas Institutional Review Board approved all study consent forms, instruments, and procedures (Study No. 00004235).

All potential survey participants were screened with the question, “Do you have a physical or mental condition, impairment, or disability that affects your daily activities and/or that requires you to use special equipment or devices, such as a wheelchair, walker, TDD [telecommunications device for the deaf] or communication device?” Those who answered “no” to this initial question were screened out, and those who answered “yes” were invited to complete the full survey, which included additional disability-related questions throughout. The sample was limited to respondents who answered all disability questions in the 2019 NSHD (*n* = 2,164).

### MEASURES

Online appendix A^22^ describes the various disability questions included in the NSHD, which included the ACS-6, the WG-SS, a self-categorized primary disability type item (intellectual or cognitive, mental illness or psychiatric, physical or mobility, chronic illness or disease, sensory, developmental, and neurological), an open-ended description of disability or health condition and age of onset, and whether or not the disability or health condition had lasted more than one year. Self-categorized disability type was used to explore the effectiveness of the ACS-6 and WG-SS question sets.

Although the ACS-6 and WG-SS questions overlap, there are two important differences. First, the WG-SS includes a question about communication difficulties, whereas the ACS-6 includes a question about performing instrumental activities of daily living (for example, errands). Second, the ACS-6 questions have dichotomous yes or no answers, whereas the WG-SS uses a scale of difficulty from 1 (no difficulty) to 4 (cannot do at all). Per Washington Group guidance, responses of “3 = a lot of difficulty” or “4 = cannot do at all” are interpreted as having a disability (see appendix A).^22^

### DATA ANALYSES

We used descriptive statistics in SPSS, version 24, to explore responses to the disability questions in the 2019 NSHD.

### LIMITATIONS

The NSHD recruitment and online data collection methods present limitations to the study and reduce generalizability. The sample was more educated and female than the US adult population with disabilities, and it likely excluded many respondents without consistent internet access, as well as people with severe intellectual and developmental disabilities. In addition, the NSHD focused on the working-age population, excluding people ages sixty-five and older.

## Study Results

Exhibit 1 presents basic demographic information for NSHD respondents by self-reported primary disability category. Across the entire sample, 98.9 percent of respondents answered “yes” to the question, “Do you currently have a health condition that has lasted for a year or more or is expected to last for a year or more?” Additionally, subtracting respondents’ self-reported age of disability onset from their current age indicated that 99.4 percent had had their disabilities for at least one year. These data indicate that the disabling conditions were not transient or related to short-term injury.

### FULL FALSE NEGATIVES

Because all respondents screened as having a disability before taking the survey, those not captured by the ACS-6 or WG-SS were considered full false negatives. Exhibit 2 shows the percentages of respondents by each self-reported disability category who answered “no” to all of theACS-6questions. Exhibit 3 shows percentages of respondents by each self-reported NSHD disability category who answered “no difficulty” (broad definition of WG-SS disability) and who answered “no difficulty” or “some difficulty” (restricted definition of WG-SS disability, recommended by the Washington Group) to all WG-SS questions.

For the ACS-6, the overall full false-negative rate for having a disability was 19.5 percent, with 422 respondents answering “no” to all six questions (exhibit 2). Those self-categorizing their disability as a chronic illness had the highest rate of full false negatives (31.6 percent), followed by those with mental illness or a psychiatric condition (22.7 percent). For the WG-SS, the full false-negative rate for the broad definition was 4.4 percent, with 96 reporting “no difficulty,” including 7.5 percent of people self-categorizing as having mental illness and 7.2 percent self-categorizing as having chronic illness (exhibit 3). This rate balloons using the WG-SS recommended definition of disability, where 43.1 percent reported either “no difficulty” or only “some difficulty” on all six WG-SS items, including 58.7 percent self-categorizing as having mental illness and 53.4 percent self-categorizing as having chronic illness.

### PARTIAL FALSE NEGATIVES

We refer to “partial false negatives” for instances in which a person responded “yes” to only an ACS-6 or WG-SS question not related to their primary self-categorized disability (for example, a person with a mental illness endorsing only the “hearing” question). Neither the ACS-6 nor the WG-SS questions were designed to identify specific conditions or disability types.^7,23^ Nevertheless, certain conditions or disability types can reasonably be expected to be associated with specific functional difficulties. The ACS-6 and WG-SS questions that would be expected to elicit positive responses for particular disability categories are indicated in exhibits 2 and 3 (for example, ACS “seeing” or “hearing” questions for those self-categorizing with a sensory disability, and WG-SS “walking” question for those self-categorizing with a physical or mobility disability). Notably, no specific functional questions from the ACS-6 or WG-SS correspond directly to people self-categorizing with chronic illnesses, neurological conditions, or developmental disabilities. Thus, in addition to many people being fully missed by the ACS-6 and WG-SS as full false negatives, an additional group is identified only in a functional category that does not correspond to their self-categorized main disability type.

## Discussion

Overall, the ACS-6 failed to identify almost one of five people (19.5 percent) in a national sample of working-age adults with self-identified, enduring disabilities. People with chronic illnesses or psychiatric disabilities were most likely to be unidentified. We classified these instances of unidentified people as full false negatives, in that no disability was indicated using the ACS-6. Further, some affirmative responses to the ACS-6 were not reflective of a person’s self-identified “main” or primary disability in the NSHD. For example, 130 (22 percent) of 589 positive responses to the ACS-6 among people self-categorizing their primary disability as physical or mobility did not respond “yes” to the ACS ambulatory question but did respond “yes” to another ACS question. Although these people would be counted in national disability reporting, their primary disability (that is, physical or mobility) would be missed. We refer to these instances as partial false negatives. For the purposes of allocating resources and understanding the potential causes and correlates of health disparities, we consider these instances to be as problematic as the full false negatives.

Using the WG-SS questions resulted in similar issues. If a “yes” response to any level of difficulty is counted, these questions capture a much higher percentage of the sample of people with disabilities, resulting in a full false-negative rate of only 4.4 percent. If responses are limited to “a lot of difficulty” or “unable to do at all,” as intended by its developers,^23^ the WG-SS false-negative rate increases dramatically to 43.1 percent. In addition, because the WG-SS questions are largely limited to the same broad functional categories as the ACS-6, issues related to partial false negatives are similar. A large percentage of the disabilities captured by the WG-SS are different from respondents’ self-categorization of their primary disability. Indeed, it was noted by the WG-SS developers^14^ that the short set would not capture the total population of people with limitations and that respondents would not represent the “true” population of people with disabilities because to capture them would require a much larger and more extensive set of questions. Whether the broad or more restrictive application of the WG-SS is used, the questions miss some populations disproportionately and lack specificity to identify particular types of disability (partial false negatives)—information that is essential for program funding decisions and disparities research.

Past research found that the ACS-6 questions tend to capture people with transitory disabilities.^11,12^ This was not the case for the NSHD sample and questions. Nearly all respondents reported having long-term disabilities, suggesting that the survey’s initial disability screening question effectively identified people with enduring disabilities. Although this result may be partially explained by the recruitment methods, which used organizations or groups that primarily work with people who have enduring or long-term disabilities, respondents recruited through Amazon’s Mechanical Turk, a population-based sample with many respondents who are not associated with disability organizations, also reported enduring disabilities. This finding makes the case for including an additional screening question in population surveys to distinguish people with enduring disability.

Understanding the prevalence and experiences of all people with disabilities is important in planning for services and supports.

In addition, our study found problems with the ACS-6 and WG-SS in capturing people with long-term disabilities, particularly those with psychiatric disabilities and those with chronic illness. Within the NSHD sample, both the ACS-6 and WG-SS had high rates of full and partial false negatives for people with long-term disabilities. Both sets of questions also rely on respondents to report “difficulty” with a task. If a respondent does not use stairs or does not have unmet needs in bathing or dressing, they might not report “difficulty” with these tasks.

Another study documented similar rates of false negatives when using a disability screening question taken from a previous version of the national Behavioral Risk Factor Surveillance System that focused on being “limited” in activities.^24^ Again, people with disabilities who have appropriate services and supports might not consider themselves to be “limited”^25^ but might report that they have a physical or mental condition, impairment, or disability that affects their daily activities, as did all respondents to the NSHD. Other researchers have also noted the problematic nature of using function-based questions such as the ACS-6 and the associated lack of accuracy and precision.^9–12^ Even if people with disabilities report no functional limitations because they have adequate services and supports, it is still essential that they be counted and their disabilities known so that those services and supports continue to be funded and the disparities continue to be documented.

Moreover, the ACS-6 and WG-SS are limited to a few categories of functioning and activities, yet the International Classification of Functioning, Disability and Health includes hundreds of such categories.^3^ The fact that the WG-SS and ACS-6 miss many people with mental and chronic illnesses, as well as some with other conditions, reflects the functional and activity areas chosen for inclusion in the question sets instead of indicating that the missed conditions do not affect daily life activities.

## Policy Implications

Two of the stated purposes of the ACS-6 are to understand the disability population and to identify “vulnerable populations that may be at disproportionate risk of experiencing limitations in health care access, poor health quality, and suboptimal health outcomes.”^2^ Our findings indicate that neither purpose is being fully achieved. For example, people with mental illness experience many poor health outcomes, with high odds for premature mortality and multiple comorbid chronic health conditions,^26,27^ but they are systematically undercounted using ACS-6 questions. Similarly, people with chronic illness are at risk for poor outcomes, including premature death, hospitalization, and lost productivity,^28^ but the types of functioning included in the ACS-6 appear to miss the difficulties that many with chronic illness experience.

Documentation for the WG-SS disability questions indicates that they should be used for two reasons: to estimate the prevalence of disability and to measure exclusion (for example, in school or employment).^23^ Although our study showed that the WG-SS resulted in full and partial false negatives for all disability categories, rates were especially high for people with mental illness. The Washington Group notes that the short-set questions fail to identify about half of people with psychosocial disabilities in the US; in our study even the broader WG-SS measure had the highest rate of false negatives for people with mentalillness.^23^ By missing a substantial proportion of this population, the questions do not provide accurate estimates of the prevalence of mental health disabilities. Further, because the WG-SS misses many people with mental illness, measures of societal exclusion are likely underestimated by surveys using the WG-SS, given that people with mental illness have high rates of exclusion from the workforce and educational opportunities.^27^

From a health policy perspective, understanding the prevalence and experiences of all people with disabilities is extremely important in planning for services and supports. For example, on a per capita basis, people with disabilities are the highest-cost population in state Medicaid programs.^29^ In addition, half of adults eligible for Medicaid via a disability determination have a mental health diagnosis.^30^ Indeed, Charles Roehrig^31^ found in 2016 that mental illness was the single most costly condition in the US, followed by a variety of chronic conditions including heart and pulmonary conditions, arthritis, and diabetes.

Our analysis showed that the ACS-6 and WG-SS questions failed to identify many people with mental illness that were classified as severe by the World Health Organization,^32^ including those who wrote schizophrenia or schizoaffective disorder, bipolar disorder, and moderate or severe depression in response to the NSHD open-ended question. The ACS-6 and the WG-SS also failed to identify many who wrote in serious chronic health conditions such as diabetes, congestive heart failure, and multiple sclerosis, even though all of these respondents self-identified as having a condition that affected their daily life activities. Just as surveys must be culturally sensitive to other marginalized groups,^33^ survey questions aimed at identifying people with disabilities should use appropriate language and provide response options that are inclusive of a large range of conditions and experiences so that respondents will not be excluded. Indeed, many respondents to the NSHD thanked us for including their perspectives and said that they often felt excluded by other surveys.^34^

The US population of people with disabilities is large and growing.^35,36^ One of the newest causes of disability is infection with the novel SARS-CoV-2 coronavirus, which can result in a spectrum of long COVID conditions for about a third of people who are infected, including a range of chronic illnesses or psychiatric and neurological symptoms.^16,17^ Although many people experiencing long COVID will likely qualify for federal disability benefits, be high users of health care, experience limitations in daily activities, or require workplace accommodations, federal surveys using only the current ACS-6 or WG-SS questions will likely significantly undercount them.

When HHS designated the ACS-6 as the federal standard disability questions, it stressed that these are the minimum data standards, with agencies permitted to include as many additional questions as desired.^5^ Further, the International Classification of Functioning, Disability and Health stresses that the social model alone is not sufficient to fully understand disability and that medical and body-level issues are also important considerations.^3^ Based on other research and our findings here, we strongly recommend that federal surveys include three additional disability questions, with additional field testing and validation. The first question should simply ask whether the respondent has a mental or physical condition, impairment, or disability that affects daily activities or requires use of equipment or technology. This broad question would capture a large sample, to be refined with subsequent questions. The second should ask what the condition or conditions are and which is the main or primary condition (via either open-ended or self-categorization questions). This question would help clarify and differentiate disability types for specific public health planning. The third should ask either age of onset, duration, or expected duration of the condition to address concerns about enduring versus transitory disability.

Asking these questions would address many of the issues related to full or partial false negatives and lack of specificity reported in the ACS-6 or WG-SS. Although we acknowledge that additional questions, especially open-ended ones, would result in additional costs and response times, an argument can be made that society cannot truly afford the costs of not capturing the added information. Algorithms could be developed to categorize most responses, and the data available to researchers and policy makers would be infinitely more inclusive and informative.

## Conclusion

The findings from our study of federal surveys suggest that many people with psychiatric disabilities and chronic illnesses are not included in national estimates of disability. Thus, public funding for these populations may also be inappropriately low. In addition, broad functional categories, such as those used in the ACS-6 and WG-SS, that do not align with self-categorized primary disabilities add complexities to reporting. In the COVID-19 pandemic, people with many chronic illnesses and disabilities are at increased risk for adverse outcomes, yet understanding of the true prevalence of these conditions may be lacking. In addition, large numbers of people with long COVID may be overlooked.

If both function- and condition-specific questions are included in these surveys, data can be used more reliably by researchers, policy makers, and practitioners to track prevalence and types of disabilities, create more supportive services and environments, understand health disparities, and address risks. Mounting evidence suggests that understanding and tracking disability at the national level can be improved, and the addition of a small complement of disability questions seems like a reasonable charge. ▪

## Supplementary Material

Supplemental_Appendix

## Figures and Tables

**Exhibit 1:** Demographic characteristics of respondents to the National Survey on Health and Disability (NSHD), by self-categorized primary disability type, 2019

	NHSD self-categorized primary disability types^a^							
		
Demographic characteristics	Physical or mobility (*n* = 589)	Mental illness (*n* = 572)	Chronic illness (*n* = 526)	Neurological (*n* = 235)	Sensory (*n* = 93)	Developmental (*n* = 86)	Intellectual or cognitive (*n* = 63)	Total (*N* = 2,164)
Gender, %								
Female	64.3	64.2	71.3	62.1	55.9	50.0	49.2	64.4
Male	34.3	32.7	25.3	34.9	40.9	39.5	47.6	32.6
Other^b^	1.4	3.1	3.4	3.0	3.2	10.5	3.2	3.0
Race and ethnicity, %								
White non-Hispanic	81.8	78.8	81.2	72.8	76.3	67.4	77.8	79
Black non-Hispanic	2.5	6.1	4.9	6.4	6.5	5.8	0.0	4.7
Other race non-Hispanic	12.1	12.8	11.8	17.4	11.8	23.3	17.4	13.3
Hispanic, all races	3.6	2.3	2.1	3.4	5.4	3.5	4.8	3.0
Age, years								
Mean	46.2	37.0	43.3	42.7	42.2	33.8	35.9	41.7
SD	12.4	11.5	12.5	12.4	12.4	11.2	12.5	12.7
Range	18–64	18–64	19–64	18–64	20–64	19–61	18–64	18–64
Age of disability onset, years								
Mean	16.8	19.9	15.9	22.4	6.4	2.9	9.0	18.8
SD	10.3	18.6	15.9	16.0	11.9	8.6	14.4	16.0
Range	0–61	0–62	0–61	0–62	0–52	0–41	0–60	0–62
Education level, % with college degree	50.4	43.8	48.9	50.6	59.1	52.3	20.6	47.9
Employment status, %								
Not employed	40.2	33.6	35.7	49.4	24.7	41.9	39.7	37.8
Employed part time	27.0	31.3	31.2	22.1	32.3	40.7	34.9	29.6
Employed full time	32.8	35.1	33.1	28.5	43.0	17.4	25.4	32.6
Income level <138% FPL, %	36.4	40.6	32.5	37.5	28.6	50.0	45.2	37.1
Population density rural,^c^ %	18.8	15.6	18.4	17.1	19.6	16.5	11.1	17.4

**SOURCE** Authors’ analysis of data from the 2019 NSHD. **NOTE** FPL is federal poverty level.

a Based on this NSHD question: “Of the options listed below which ONE category would you use to describe your main disability or health condition?” with the order of the 7 options listed randomized.

b Includes nonbinary, transgender, gender nonconforming, genderqueer, agender, two-spirit, intersex, and so on, as written in by respondents.

c County of residence had a population density of fewer than 50,000 people (micropolitan and noncore categories), using county-level rural-urban commuting area codes.

**Exhibit 2:** National Survey on Health and Disability (NSHD) respondents’ self-categorized primary disability types by American Community Survey 6 (ACS-6) item, 2019

NSHD self-categorized primary disability types^a^	Positive response to ACS-6 disability questions						Negative response to all ACS-6 disability questions

	Seeing	Hearing	Concentrating	Walking	IADLs	ADLs	
Physical or mobility disability	10.7%	8.1%	26.5%	77.9%^b^	45.8%	49.4%	12.1%
Mental illness or psychiatric	4.4	4.7	65.6^b^	13.6	8.6	42.8	22.7
Chronic illness or disease	6.7	5.9	40.7	40.5	21.3	37.1	31.6
Neurological	12.8	7.2	56.6	49.4	29.8	48.1	14.0
Sensory	45.2^b^	46.2^b^	17.2	10.8	3.2	26.9	3.2
Developmental	11.6	9.3	55.8	37.2	31.4	52.3	11.6
Intellectual or cognitive	14.3	15.9	73.0^b^	22.2	27.0	61.9	12.7
Total	9.9	8.5	45.7	42.6	25.3	44.0	19.5

**SOURCE** Authors’ analysis of data from the 2019 NSHD. **NOTES** Numbers of respondents by disability type are in exhibit 1. Question responses are dichotomous (yes/no). IADL is instrumental activities of daily living. ADL is activities of daily living.

a Categories in this column are derived from this NSHD survey question: “Of the options listed below which ONE category would you use to describe your main disability or health condition?” with the order of the 7 options listed randomized. The ACS-6 questions read as follows: Are you blind or do you have serious difficulty seeing even when wearing glasses? Are you deaf or do you have serious difficulty hearing? Because of a physical, mental, or emotional condition, do you have serious difficulty concentrating, remembering, or making decisions? Do you have serious difficulty walking or climbing stairs? Do you have difficulty bathing or dressing? Because of a physical, mental, or emotional condition, do you have difficulty doing errands alone, such as visiting a doctor’s office or shopping?

b A positive response would be expected to this disability question for the particular disability category shown.

**Exhibit 3:** National Survey on Health and Disability (NSHD) respondents’ self-categorized primary disability types, by Washington Group Short Set (WG-SS) item, broad and restricted definitions, 2019

NSHD self-categorized primary disability types^a^	Positive responses to individual WG-SS disability questions						Negative response to all WG-SS disability questions^b^

	Seeing	Hearing	Remembering or concentrating	Walking	Self-care	Communicating	
**BROAD DEFINITION** ^c^							
Physical or mobility disability	54.2%	26.8%	59.5%	92.3%^d^	71.8%	22.6%	1.4%
Mental illness or psychiatric	47.9	20.3	86.2^b^	35.7	30.1	32.7	7.5
Chronic illness or disease	54.2	29.7	75.8	77.1	36.9	25.9	7.2
Neurological	57.4	27.2	84.3	72.8	49.4	46.0	1.3
Sensory	74.2^c^	59.1^c^	46.2	34.4	10.8	32.3	1.1
Developmental	45.3	29.1	73.3	66.3	57.0	65.1	2.3
Intellectual or cognitive	49.2	33.3	93.7^c^	50.8	49.2	73.0	3.2
Total	53.9	27.6	73.9	63.5	43.0	32.3	4.4
**RESTRICTED DEFINITION** ^e^							
Physical or mobility disability	8.7%	4.2%	13.1%	62.3%^d^	21.6%	0.7%	26.3%
Mental illness or psychiatric	4.5	3.0	27.6^c^	7.5	6.1	3.3	58.7
Chronic illness or disease	5.1	2.9	24.1	25.1	7.4	1.3	53.4
Neurological	13.6	5.1	34.0	38.7	18.3	2.1	37.0
Sensory	40.9^c^	34.4^c^	5.4	6.5	0.0	2.2	24.7
Developmental	7.0	5.8	32.6	31.4	22.1	4.7	34.9
Intellectual or cognitive	7.9	11.1	46.0^c^	14.3	17.5	11.1	33.3
Total	8.5	5.2	23.3	31.2	12.7	2.2	43.1

**SOURCE** Authors’ analysis of data from the 2019 NSHD. **NOTE** Numbers of respondents by disability type are in exhibit 1.

a Categories in this column are derived from this NSHD survey question: “Of the options listed below which ONE category would you use to describe your main disability or health condition?” with the order of the 7 options listed randomized. The WG-SS disability question reads as follows: How much difficulty do you have seeing even if wearing glasses? Hearing even if using a hearing aid? Remembering or concentrating? Walking or climbing stairs? With self-care, such as washing all over or dressing? Communicating, for example, understanding or being understood by others?

b Negative response is “no difficulty.”

c The broad definition is answering “cannot do at all,” “a lot of difficulty,” or “some difficulty” to the functional disability questions.

d A positive response would be expected to this disability question for the disability category shown.

e The restricted definition is answering “cannot do at all” or “a lot of difficulty” to the functional disability questions.
