# Supplementary material for: Comparing Measures Of Functional Difficulty With Self-Identified Disability: Implications For Health Policy
Source: Health Aff (Millwood). Author manuscript; Available in PMC 2023 Jul 18. (PMC10353341; doi:10.1377/hlthaff.2022.00395)
Supplement: Supplemental_Appendix [file NIHMS1903697-supplement-Supplemental_Appendix.pdf]

APPENDIX

Appendix A: Disability Measures Included in the 2019 National Survey on Health and Disability

**Disability Question(s) or Measure**	**Response Option(s)**	**Description**
1. Do you currently have a health condition that has lasted for a year or more or is expected to last for a year or more? This could be a physical health condition (such as arthritis, asthma, cancer, dementia, diabetes, heart disease, hypertension, or stroke), a behavioral health or mental health condition, or a developmental disability?	• Yes, one condition<br>• Yes, more than one condition<br>• No	Used since 2014 in the national Health Reform Monitoring Survey (HRMS) administered by The Urban Institute.[1]<br><br>Analyses:<br>Used to assess disability duration.
1. What is your disability and/or health condition(s)? If you have more than one, please list your main one first?<br>2. What age did your main disability or health condition begin for you?	• Open-ended responses	Two items to assess disability diagnosis and age of onset.<br><br>Analyses:<br>Used to assess disability duration
1. Which ONE category would you use to describe your main disability or health condition?	• Intellectual/cognitive<br>• Mental illness/psychiatric<br>• Physical/mobility<br>• Chronic illness or disease<br>• Sensory<br>• Developmental<br>• Neurological	Categories based on other national survey questions, recommendations from an expert panel of disability researchers, and pilot-tests with people reporting disabilities.<br><br>Analyses:<br>Used to identify groups included in full and partial false-negatives in ACS and WG-SS responses.
**ACS-6**<br>1. Are your deaf or do you have serious difficulty hearing?<br>2. Are you blind or do you have serious difficulty seeing even when wearing glasses?	• Yes<br>• No	A “no” response to all 6 questions indicates the absence of disability.<br><br>Analyses:

3. Because of a physical, mental, or emotional conditions, do you have serious difficulty concentrating, remembering, or making decisions?<br>4. Do you have serious difficulty walking or climbing stairs?<br>5. Do you have difficulty bathing or dressing?<br>6. Because of a physical, mental, or emotional condition, do you have difficulty doing errands alone, such as visiting a doctor's office or shopping?		Used to identify groups included in false and partial false negatives in ACS.
**WG-SS**<br>1. How much difficulty do you have seeing, even if wearing glasses?<br>2. How much difficulty to you have hearing even if using a hearing aid?<br>3. How much difficulty do you have remembering or concentrating?<br>4. How much difficulty do you have walking or climbing stairs?<br>5. How much difficulty do you have with self-care, such as washing all over or dressing?<br>6. How much difficulty do you have communicating, for example understanding or being understood by others.	• No difficulty<br>• Some difficulty<br>• A lot of difficulty<br>• Cannot do at all	The Washington Group suggests a classification scheme for measuring disability. For each question, those who respond "a lot of difficulty" or "cannot do at all" are considered to have functional disability in that area. A "no difficulty" or "some difficulty" response indicates the absence of a disability in each area with "no difficulty" or "some difficulty" responses to all six items indicating absence of any disability.<br><br>Analyses:<br>Used to identify groups included in full and partial false-negatives in WG-SS.

1. Urban Institute. Health Reform Monitoring Survey [Internet]. Urban Institute. 2021 [cited 2022 Feb 24]. Available from: https://www.urban.org/policy-centers/health-policy-center/projects/health-reform-monitoring-survey
